# Associations Among Maternal Metabolic Conditions, Cord Serum Leptin Levels, and Autistic Symptoms in Children

**DOI:** 10.3389/fpsyt.2021.816196

**Published:** 2022-02-03

**Authors:** Toshiki Iwabuchi, Nagahide Takahashi, Tomoko Nishimura, Md Shafiur Rahman, Taeko Harada, Akemi Okumura, Hitoshi Kuwabara, Shu Takagai, Yoko Nomura, Hideo Matsuzaki, Norio Ozaki, Kenji J. Tsuchiya

**Affiliations:** ^1^Research Center for Child Mental Development, Hamamatsu University School of Medicine, Hamamatsu, Japan; ^2^United Graduate School of Child Development, Hamamatsu University School of Medicine, Hamamatsu, Japan; ^3^Department of Child and Adolescent Psychiatry, Nagoya University Graduate School of Medicine, Nagoya, Japan; ^4^Department of Psychiatry, Nagoya University Graduate School of Medicine, Nagoya, Japan; ^5^Department of Psychiatry, Hamamatsu University School of Medicine, Hamamatsu, Japan; ^6^Department of Psychiatry, Saitama Medical University, Saitama, Japan; ^7^Department of Child and Adolescent Psychiatry, Hamamatsu University School of Medicine, Hamamatsu, Japan; ^8^Queens College and Graduate Center, City University of New York, New York City, NY, United States; ^9^Research Center for Child Mental Development, University of Fukui, Fukui, Japan; ^10^United Graduate School of Child Development, University of Fukui, Fukui, Japan

**Keywords:** autism spectrum disorder, maternal metabolic conditions, overweight, diabetes mellitus, hypertensive disorders of pregnancy, leptin

## Abstract

**Introduction:**

Accumulating evidence has shown that maternal metabolic conditions, such as pre-pregnancy overweight, diabetes mellitus, and hypertensive disorders of pregnancy (HDP) are potential risk factors of autism spectrum disorder (ASD). However, it remains unclear how these maternal conditions lead to neurodevelopmental outcomes in the offspring, including autistic symptoms. Leptin, an adipokine that has pro-inflammatory effects and affects fetal neurodevelopment, is a candidate mediator of the association between maternal metabolic factors and an increased risk of ASD. However, whether prenatal exposure to leptin mediates the association between maternal metabolic conditions and autistic symptoms in children has not been investigated yet.

**Methods:**

This study investigated the associations between mothers' metabolic conditions (pre-pregnancy overweight, diabetes mellitus during or before pregnancy, and HDP), leptin concentrations in umbilical cord serum, and autistic symptoms among 762 children from an ongoing cohort study, using generalized structural equation modeling. We used the Social Responsive Scale, Second Edition (SRS-2) at 8–9 years old to calculate total T-scores. Additionally, we used the T-scores for two subdomains: Social Communication and Interaction (SCI) and Restricted Interests and Repetitive Behavior (RRB).

**Results:**

Umbilical cord leptin levels were associated with pre-pregnancy overweight [coefficient = 1.297, 95% confidence interval (CI) 1.081–1.556, *p* = 0.005] and diabetes mellitus (coefficient = 1.574, 95% CI 1.206–2.055, *p* = 0.001). Furthermore, leptin levels were significantly associated with SRS-2 total T-scores (coefficient = 1.002, 95% CI 1.000–1.004, *p* = 0.023), SCI scores (coefficient = 1.002, 95% CI 1.000–1.004, *p* = 0.020), and RRB scores (coefficient = 1.001, 95% CI 1.000–1.003, *p* = 0.044) in children. Associations between maternal metabolic factors and autistic symptoms were not significant.

**Discussion:**

The present study uncovered an association between cord leptin levels and autistic symptoms in children, while maternal metabolic conditions did not have an evident direct influence on the outcome. These results imply that prenatal pro-inflammatory environments affected by maternal metabolic conditions may contribute to the development of autistic symptoms in children. The findings warrant further investigation into the role of leptin in the development of autistic symptoms.

## Introduction

Autism spectrum disorder (ASD) is a highly prevalent neurodevelopmental condition, with >3% prevalence in Japan (1), characterized by difficulties in social communication and repetitive and restricted patterns in behaviors and interests (2). Autistic symptoms are observable across the general population and are not limited to the clinical population (3). Although ASD is heritable (4, 5), various environmental factors are plausibly associated with an increased risk of ASD (6, 7). Moreover, previous studies have demonstrated associations between prenatal or perinatal factors and autistic symptoms in children (8). Recently, several umbrella reviews have provided an overview of the important contributions of environmental factors, including obstetric, demographic, and neurotoxic factors, on ASD (9, 10). However, the biological mechanisms that link environmental factors and elevated autistic symptoms remain largely unexplored.

Previous studies have implicated maternal metabolic conditions before and during pregnancy, including diabetes mellitus (DM) (11–13), hypertensive disorder of pregnancy (HDP) (14–17), and pre-pregnancy overweight (18–20), with an increased risk of ASD. The global burden of such metabolic conditions (21–23) warrants studies on understanding the mechanisms by which maternal factors are associated with ASD pathogenesis. Several researchers have highlighted the role of maternal metabolic condition-induced inflammation in neurodevelopmental outcomes in children (24–26). Maternal pre-pregnancy overweight or obesity has been linked to *in utero* inflammatory environments, consequently affecting fetal neurodevelopment (27, 28). Animal studies have further elaborated that maternal DM enhances the production of pro-inflammatory cytokines or chemokines, which adversely affects fetal neurodevelopment (29, 30). Likewise, the association of HDP, especially preeclampsia, with enhanced inflammation has been supported by animal studies (31, 32). However, limited evidence from human longitudinal studies challenges the identification of causal pathways between maternal metabolic conditions and ASD.

Leptin, an adipokine (or adipocytokine), has pro-inflammatory effects and may mediate the link of maternal metabolic diseases to neurodevelopmental outcomes in children (33). Leptin, primarily secreted from the white adipose tissues, plays an important role not only in the regulation of food intake by acting on the hypothalamus (34, 35) but also in immunity and inflammation (36, 37). For example, leptin upregulates the secretion of pro-inflammatory cytokines such as tumor necrosis factor (TNF)-α and interleukin (IL)-1β (38–40). Conversely, other studies have suggested that leptin production is upregulated by TNF-α and IL-1β (41–43), possibly forming a pro-inflammatory feedback loop. The administration of inflammatory stimuli, such as lipopolysaccharide, enhances leptin production (44, 45), whereas leptin deficiency likely results in an immunosuppressive phenotype characterized by reduced levels of inflammatory cytokines (46, 47). Furthermore, animal studies have suggested that maternal systemic inflammation, induced by factors such as a high-fat diet, causes disrupted leptin signaling and affects neurodevelopment in children (48–50). Collectively, these findings indicate that leptin may contribute to strengthening *in utero* neuroinflammation induced by maternal metabolic conditions and consequently influence neurodevelopment in the offspring.

An association between leptin and ASD has also been suggested previously. A postmortem study found increased leptin levels in the anterior cingulate gyrus of individuals with ASD (51). As for peripheral markers, several cross-sectional studies have reported higher serum or plasma leptin levels in individuals with ASD compared to those in typically developing individuals (52–56). A longitudinal study reported that higher plasma leptin levels in early childhood (mean age of measurement, 18.4 months) were associated with an increased risk for later diagnosis of ASD (57). Another recent study suggested that children at 4–12 years of age who received an ASD diagnosis, compared to their typically developing counterparts, showed different trajectory patterns in peripheral leptin levels (58). However, most of these studies measured leptin levels in serum or plasma samples collected from children or adults and examined their associations with ASD. Associations between umbilical cord leptin levels and later ASD symptoms have been scarcely examined except for two recent studies (57, 59). These studies examined whether cord plasma leptin levels were associated with later ASD diagnosis or autistic symptoms, but found no significant associations between them. However, the relatively small sample sizes used in these studies restricts the generalizations of their findings. Furthermore, no study has investigated whether leptin acts as a mediator of the link between maternal metabolic conditions and ASD-like behavioral characteristics in children.

Using a population-representative birth cohort (60), we aimed to examine the associations among maternal metabolic conditions (maternal diabetes, pre-pregnancy overweight, and HDP), leptin concentrations in umbilical cord serum, and later autistic symptoms in children. We hypothesized that the cord serum leptin levels mediate the link between maternal metabolic conditions and autistic symptoms. We employed path analysis to investigate (1) whether maternal metabolic conditions before or during pregnancy were associated with autistic symptoms in children of 8–9 years of age, and (2) if yes, whether the association is mediated by umbilical cord leptin levels.

## Methods

### Participants

The present study used a subsample of the Hamamatsu Birth Cohort for Mother and Child (HBC) Study, which included 762 children and their 699 mothers (see the section Results for details). The HBC Study consisted of 1,138 mothers and their children (*n* = 1,258; 611 boys, 647 girls) born in Japan between December 2007 and June 2011. Our previous study described detailed recruitment procedures (60). The present study was conducted in accordance with the Declaration of Helsinki, and written informed consent was obtained from each mother for the participation of herself and her infant. The Hamamatsu University School of Medicine and the University Hospital Ethics Committee approved the study protocol (Ref. 18-166, 19-9, 20-82, 22-29, 24-67, 24-237, 25-143, 25-283, E14-062, E14-062-1, E14-062-3, 17-037, 17-037-3, 20-233).

### Measurement

#### Autistic Symptoms

Using the Japanese version of the Social Responsive Scale, Second Edition (SRS-2) (61), we assessed autistic symptoms in children aged 8–9 years. The SRS-2 raw scores were evaluated based on the responses of a parent or caregiver to 65 items. We then converted the raw scores to T-scores normed for sex [mean = 50, standard deviation (SD) = 10]. We used the SRS-2 total T-scores as a proxy for autistic symptoms in children. Higher T-scores indicate higher ASD-like behavioral characteristics. Additionally, we used T-scores compatible with the fifth edition of the Diagnostic and Statistical Manual of Mental Disorders (DSM-5) (2), consisting of the Social Communication and Interaction (SCI) and Restricted Interests and Repetitive Behavior (RRB) scores.

#### Leptin Levels in Umbilical Cord Serum

Umbilical cord blood samples were collected from mothers immediately after delivery *via* venipuncture of the umbilical vein. The samples were kept at room temperature for 30 min after collection and then centrifuged at 3,500 rpm for 10 min, from which serum was taken and divided into 200 μl aliquots, and stored at −80°C until analysis (60). Leptin concentrations in cord serum were measured using enzyme-linked immunosorbent assay kits by Skylight Biotech, Inc. (Akita, Japan), as described previously (62). Leptin levels in cord serum ranged 0.1–78.1 ng/mL, and participants with zero value for cord serum leptin (two children) were excluded from the analysis.

#### Maternal Metabolic Conditions: Pre-pregnancy Overweight, DM Before or During Pregnancy, and HDP

Based on the pre-pregnancy body mass index (BMI), mothers were categorized as overweight (BMI ≥ 25) and non-overweight (BMI <25). Clinical diagnoses of maternal DM before or during pregnancy and HDP were evaluated based on the electronic medical records. All of them were treated as dichotomous variables.

#### Covariates

We included children's sex, maternal age at delivery, educational attainment of mothers, household income at birth, gestational age at birth (<37 or ≥37 weeks), birth weight, maternal smoking status during pregnancy, and mode of feeding during 0–6 months of age (breastfeeding only, formula only, breastfeeding, and formula) in the model as possible confounders based on previous studies (57, 63).

### Statistical Analysis

**Figure 1:**
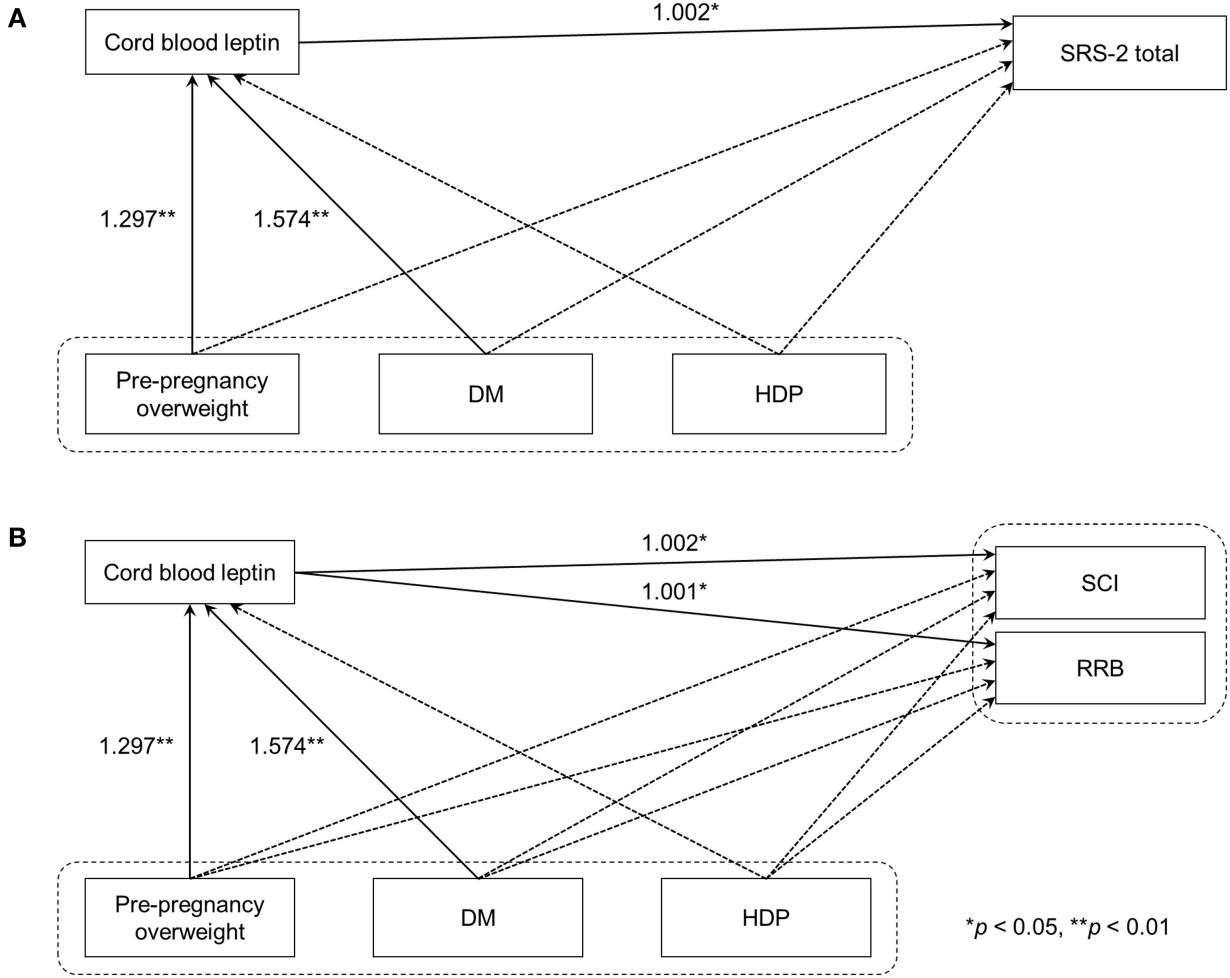
Path analyses of maternal metabolic conditions, cord blood leptin concentrations, and autistic symptoms in children. **(A)** Model 1 used SRS-2 total T-scores as the indicator of autistic symptoms in children. **(B)** In Model 2, DSM-5 compatible T-scores were used instead. Solid line arrows and dashed line arrows indicate significant and non-significant associations between observed variables, respectively. Numbers represent exponentiated coefficients obtained from the analysis (^*^p < 0.05, ^**^p < 0.01). DM, diabetes mellitus before or during pregnancy; HDP, hypertensive disorders of pregnancy; SRS-2, the Social Responsive Scale, Second Edition; DSM-5, the fifth edition of the Diagnostic and Statistical Manual of Mental Disorders; SCI, Social Communication and Interaction; RRB, Restricted Interests and Repetitive Behavior.

We conducted path analysis to investigate a series of associations between maternal metabolic conditions, leptin concentrations, and children's autistic symptoms. The data on leptin levels in cord serum and SRS-2 scores (i.e., total, SCI, and RRB scores) were not normally distributed (all *p* < 0.001, Shapiro-Wilk tests). Since the non-negative data (i.e., SRS-2 T-scores and leptin levels in cord serum) had positively skewed distributions, we employed a generalized structural equation modeling with gamma family and log link (64–67). The model was adjusted for the covariates mentioned above. We used the *gsem* command in Stata version 15.1 to perform the path analysis. First, we performed path analysis using SRS-2 total T-scores to indicate autistic symptoms (Model 1; Figure 1A). Second, if SRS-2 total scores were significantly associated with any variable, we investigated whether the umbilical cord leptin levels and maternal metabolic conditions were associated with autistic symptoms in two subdomains compatible with DSM-5 (i.e., SCI and RRB; Model 2; Figure 1B). When the *gsem* command is used, full-information maximum likelihood (FIML) estimation is not available to deal with missing data. Therefore, we conducted sensitivity analyses using the *sem* command with the FIML option, although non-normality was not considered. All participants in the HBC Study were included in these analyses.

## Results

### Participant Characteristics

A total of 840 participants who completed the SRS-2 assessment were included in the analysis. Of them, 78 children were excluded due to the lack of data on cord blood leptin levels, leaving 762 children and 699 mothers included in the analysis. The participant characteristics are summarized in Table 1. Using Little's test with the *mcartest* command in Stata (68, 69), we confirmed that the missingness of the variables of interest included in the model was completely at random (χ^2^ = 9.74, *df* = 16, *p* = 0.87) and not dependent on covariates (χ^2^ = 162.23, *df* = 160, *p* = 0.43).

**Table 1:** Sample characteristics of participating children and their parents.

	***n* (%)**
Child's sex	
Male	386 (50.7)
Female	376 (49.3)
Preterm birth	44 (5.8)
Mother's smoking status during pregnancy	55 (7.2)
Mode of feeding	
Breastfeeding only	424 (33.7)
Formula only	237 (18.8)
Breastfeeding and formula	597 (47.5)
Maternal pre-pregnancy overweight	75 (9.8)
Diabetes mellitus before or during pregnancy	8 (1.0)
Hypertensive disorders of pregnancy	87 (11.4)
	**Mean (SD)**
Leptin levels in cord serum (ng/mL)	4.5 (5.3)
Birth weight (g)	2945.5 (432.7)
Gestational age at birth (weeks)	39.0 (1.5)
Maternal age at birth (years)	31.9 (4.9)
Household income at birth (million JPY)	6.2 (2.8)
Mother's education (years)	14.0 (1.8)
SRS-2 T-scores	
Total	51.2 (7.3)
Social Communication and Interaction (SCI)	53.9 (9.0)
Restricted Interests and Repetitive Behavior (RRB)	49.6 (7.4)

*SD, Standard Deviation; JPY, Japanese yen; SRS-2, the Social Responsiveness Scale, Second Edition*.

### Associations Among Maternal Metabolic Conditions, Cord Blood Leptin, and Autistic Symptoms

As the coefficients were log-transformed, we present the exponentiated coefficients hereafter. In both Model 1 and Model 2, maternal pre-pregnancy overweight [Model 1, coefficient = 1.297, 95% confidence interval (CI) 1.081–1.556, *p* = 0.005; Model 2, coefficient = 1.297, 95% CI 1.081–1.556, *p* = 0.005] and DM (Model 1, coefficient = 1.574, 95% CI 1.206–2.055, *p* = 0.001; Model 2, coefficient = 1.574, 95% CI 1.206–2.055, *p* = 0.001) were significantly associated with increased levels of leptin in cord serum. In Model 1, we found that cord leptin concentrations were positively associated with SRS-2 total T-scores (coefficient = 1.002, 95% CI 1.000–1.004, *p* = 0.023; Table 2). This result indicates that a one-unit increase in cord serum leptin multiplies the SRS-2 total T-score by 1.002. As for the covariates, cord leptin levels were associated with children's sex (higher in female, coefficient = 0.549, 95% CI 0.493–0.612, *p* < 0.001), maternal age at delivery (coefficient = 0.984, 95% CI 0.973–0.995, *p* = 0.005), gestational age at birth (coefficient = 0.699, 95% CI 0.538–0.909, *p* = 0.008), and birth weight (coefficient = 1.001, 95% CI 1.000–1.001, *p* < 0.001) (see Supplementary Table 1 for details). Moreover, the additional analysis (Model 2) revealed that cord serum leptin concentrations were significantly associated with both SCI scores (coefficient = 1.002, 95% CI 1.000–1.004, *p* = 0.020) and RRB scores (coefficient = 1.001, 95% CI 1.000–1.003, *p* = 0.044). We found no direct associations between maternal metabolic conditions (i.e., maternal pre-pregnancy overweight, DM, or HDP) and autistic symptoms in children (i.e., total, SCI, and RRB scores) (all *p* > 0.05; Tables 2, 3). The sensitivity analyses confirmed that the associations mentioned above remained significant even when missing values were handled with FIML (Tables 4, 5).

**Table 2:** Estimated coefficients and *p*-values in Model 1.

**Outcome**	**Exposure**	**Coefficient (95% CI)^a^**	***P*-value**
SRS-2 total	Leptin level	**1.002 (1.000-1.004)**	**0.023**
	Pre-pregnancy overweight	1.000 (0.967-1.035)	0.956
	DM	0.997 (0.949-1.049)	0.934
	HDP	1.008 (0.977-1.039)	0.614
Leptin level	Pre-pregnancy overweight	**1.297 (1.081-1.556)**	**0.005**
	DM	**1.574 (1.206-2.055)**	**0.001**
	HDP	0.983 (0.832-1.163)	0.848

a *Coefficients are exponentiated*.

*SRS-2, the Social Responsiveness Scale, Second Edition; DM, diabetes mellitus before or during pregnancy; HDP, hypertensive disorders of pregnancy. The bold values indicate statistical significance*.

**Table 3:** Estimated coefficients and *p*-values in Model 2.

**Outcome**	**Exposure**	**Coefficient (95% CI)^a^**	***P*-value**
SCI	Leptin level	**1.002 (1.000-1.004)**	**0.020**
	Pre-pregnancy overweight	1.001 (0.963-1.041)	0.934
	DM	0.999 (0.942-1.059)	0.987
	HDP	1.008 (0.972-1.045)	0.654
RRB	Leptin level	**1.001 (1.000-1.003)**	**0.044**
	Pre-pregnancy overweight	1.021 (0.987-1.057)	0.215
	DM	1.023 (0.972-1.077)	0.370
	HDP	1.009 (0.977-1.041)	0.564
Leptin level	Pre-pregnancy overweight	**1.297 (1.081-1.556)**	**0.005**
	DM	**1.574 (1.206-2.055)**	**0.001**
	HDP	0.983 (0.832-1.163)	0.848

a *Coefficients are exponentiated*.

*SCI, Social Communication and Interaction; RRB, Restricted Interests and Repetitive Behavior; DM, diabetes mellitus before or during pregnancy; HDP, hypertensive disorders of pregnancy. The bold values indicate statistical significance*.

**Table 4:** Result of the sensitivity analysis (Model 1).

**Outcome**	**Exposure**	**Coefficient (95% CI)^a^**	***P*-value**
SRS-2 total	Leptin level	**0.086 (0.012-0.159)**	**0.022**
	Pre-pregnancy overweight	0.005 (−0.063-0.074)	0.883
	DM	−0.015 (−0.084-0.053)	0.656
	HDP	0.000 (−0.067-0.069)	0.983
Leptin level	Pre-pregnancy overweight	**0.083 (0.029-0.137)**	**0.003**
	DM	**0.165 (0.111-0.218)**	**<0.001**
	HDP	−0.027 (−0.080-0.024)	0.302

a *Standardized coefficients are shown*.

*SRS-2, the Social Responsiveness Scale, Second Edition; DM, diabetes mellitus before or during pregnancy; HDP, hypertensive disorders of pregnancy. The bold values indicate statistical significance*.

**Table 5:** Result of the sensitivity analysis (Model 2).

**Outcome**	**Exposure**	**Coefficient (95% CI)^a^**	***P*-value**
SCI	Leptin level	**0.094 (0.020-0.167)**	**0.012**
	Pre-pregnancy overweight	0.007 (−0.061-0.075)	0.839
	DM	−0.014 (−0.083-0.054)	0.677
	HDP	−0.001 (−0.069-0.067)	0.972
RRB	Leptin level	**0.082 (0.008-0.155)**	**0.028**
	Pre-pregnancy overweight	0.047 (−0.020-0.116)	0.171
	DM	0.017 (−0.050-0.086)	0.613
	HDP	0.006 (−0.061-0.075)	0.847
Leptin level	Pre-pregnancy overweight	**0.083 (0.029-0.137)**	**0.003**
	DM	**0.164 (0.110-0.218)**	**<0.001**
	HDP	−0.028 (−0.080-0.024)	0.290

a *Standardized coefficients are shown*.

*SCI, Social Communication and Interaction; RRB, Restricted Interests and Repetitive Behavior; DM, diabetes mellitus before or during pregnancy; HDP, hypertensive disorders of pregnancy. The bold values indicate statistical significance*.

## Discussion

Using longitudinal data from a population-representative birth cohort in Japan, the present study examined associations among maternal metabolic conditions, umbilical cord serum leptin levels, and autistic symptoms in children aged 8–9 years. The results demonstrated that maternal DM and pre-pregnancy overweight were associated with leptin levels in cord serum. As expected, we found significant associations between cord serum leptin levels and increased autistic symptoms indexed by SRS-2 total T-scores. Additionally, the path analysis showed that cord serum leptin levels were associated with both impaired social communication (measured in SCI) and restricted interests and repetitive behavior (measured in RRB), indicating cord leptin level as a biological factor associated with the two subdomains of ASD symptoms in common. In contrast, we found no significant associations between maternal metabolic conditions (pre-pregnancy overweight, DM, and HDP) and autistic symptoms in children. This non-significant association is inconsistent with our hypothesis that maternal metabolic conditions are linked to later autistic symptoms.

To the best of our knowledge, the present study is the first to identify an association between leptin levels in umbilical cord serum and later autistic symptoms in children. Only two studies have examined associations between adipokine levels in cord plasma and ASD (57, 59), reporting no significant association of cord leptin levels with a later ASD diagnosis and autistic symptoms. There are some methodological differences between these studies and the present study. For example, Raghavan et al. (57) reported no significant association between leptin concentrations in cord plasma and ASD diagnosis [odds ratio (OR) = 0.90, 95% CI 0.66–1.24]. However, the study by Raghavan et al. did not investigate an association between leptin and autistic symptoms observed across the general population, and the number of children receiving ASD diagnosis was small in that study (39 out of 655 children with cord blood samples). The study by Joung et al. (59) examined associations between autistic symptoms assessed using SRS-2 and cord leptin levels, similar to the present study, but reported no significant association between them (β = −0.20, 95% CI −1.34–0.94). However, the sample size (295 children) was smaller than that in our study. Needless to mention that further investigations are needed, the present findings underscore the importance of cord leptin levels in altered neurodevelopment associated with later autistic symptoms.

Several biological pathways possibly link increased cord leptin levels and increased autistic symptoms at a later stage of development. As mentioned earlier, increased levels of leptin in cord blood may reflect pro-inflammatory prenatal environments (33). Recently, *in utero* inflammation has garnered attention as a potential risk for altered neurodevelopment, which may lead to various psychiatric disorders, including ASD (70). Because leptin can cross the blood-brain barrier (71), leptin levels in cord serum may reflect elevated leptin levels in the fetal brain and increased neuroinflammation modulated by leptin. Other possible pathways may involve mitochondrial dysfunctions and/or oxidative stress (72, 73), both of which have been associated with ASD (74, 75), possibly resulting from *in utero* leptin exposure. Multiple studies have reported several other important roles of leptin in neurodevelopment. For example, leptin-deficient animals showed alterations in neuronal and cortical development (76, 77) and myelination (78). A recent study demonstrated that leptin is also associated with the emergence of inhibitory function of GABAergic neurons (79). Given the repeated observations of altered cortical structures (80–82), reduced white matter integrity (83, 84), and excitatory/inhibitory imbalance (85, 86) in ASD, it seems plausible to assume that leptin levels at birth play an important role in ASD etiopathology.

Contrary to our hypothesis, we did not find any significant association between maternal metabolic conditions and autistic symptoms although maternal overweight/obesity and DM are relatively well-established risk factors for ASD (9). One possible explanation for this discrepancy is the difference in ethnic populations. In previous meta-analyses or systematic reviews, most of the included studies were conducted in Western countries; for example, in a recent meta-analysis on the association between maternal BMI and children's ASD included studies from the USA or European countries exclusively (20). However, Asian children, including Japanese children, have lower birth weights than their Western peers (87). Higher birth weight is generally associated with higher levels of cord blood leptin (88, 89); therefore, cord blood leptin concentrations may be lower in Japanese children than in children born in other countries. The present study demonstrated that maternal DM and overweight were associated with elevated leptin levels in cord blood, and that increased leptin levels were associated with increased autistic symptoms in children. For children with relatively higher birth weights and higher cord serum leptin levels at birth, the effects of maternal metabolic conditions on perinatal leptin levels may be more severe on later neurodevelopment. In contrast, children in countries with relatively lower birth weights and baseline leptin levels (e.g., children in Japan) may be less predisposed to an increased risk of ASD attributable to maternal metabolic conditions. Taken together, we speculate that the effects of maternal metabolic conditions on autistic symptoms are less prominent in Japanese children than in those of other ethnicities. Future studies are required to clarify the reasons for these inconsistent results.

The present study replicated the previous findings that maternal DM and pre-pregnancy overweight increased cord blood leptin concentrations (90–95). In adults, blood leptin levels correlate with BMI (96, 97), and hyperleptinemia and leptin resistance are prevalent in individuals with obesity or overweight (98, 99); this is also the case for pregnant women (100). Moreover, during pregnancy, leptin is supplied not only by maternal adipose tissues but also by the placenta (101). While it is still inconclusive whether metabolic conditions of mothers (such as obesity) upregulate or downregulate the placental production of leptin (102), DM and pre-pregnancy overweight likely cause an increase in cord serum leptin levels, thereby leading to altered neurodevelopment in offspring.

No significant association was observed between HDP and cord serum levels of leptin. This does not corroborate previous studies reporting significant associations between HDP and elevated leptin levels (103–105). However, most of these studies compared mothers with preeclampsia and those without hypertensive disorders. By definition, HDP consists of several clinical conditions, such as chronic hypertension and preeclampsia (106); such heterogeneity possibly resulted in this discrepancy. Further studies with larger sample sizes would help resolve this inconsistency.

The present study has several limitations. First, the sample size of the present study was relatively small, given that the number of mothers who had metabolic conditions, especially DM (only 1.0%), was very low. This may have affected the negative findings of the associations between these conditions and autistic symptoms. In addition, although we found a significant association between cord serum leptin levels and autistic symptoms, the effect size in the present study was small compared to other studies like Raghavan et al. (57), which showed an association between early childhood plasma leptin levels and later ASD diagnosis (OR = 1.80, 95% CI = 1.25–2.60, *p* = 0.002). We believe the smaller effect size in our study compared to that in Raghavan et al.'s is primarily due to differences in the outcomes being measured (ASD symptoms vs. ASD diagnosis), but further replication with larger sample sizes is needed. Second, our analysis included only a subsample of the population-representative HBC Study. Although we confirmed that the missingness of outcome variables was completely at random and independent of the covariates, caution should be exercised when generalizing the present findings. Third, we considered maternal DM before or during pregnancy and HDP as categorical, although these diseases consisted of distinguishable clinical conditions; for example, gestational diabetes and pre-pregnancy DM can be differentiated, but these were considered as one condition in the present study. Future studies should investigate the effects of these conditions separately. Fourth, we did not confirm ASD diagnosis but relied on parental reports to evaluate autistic symptoms in children. Although SRS-2 is a validated and reliable measure, associations among maternal metabolic conditions, cord leptin levels, and clinical diagnosis of ASD in children must be further investigated.

The present study examined a series of associations among maternal metabolic conditions, umbilical cord serum levels of leptin, and autistic symptoms in children aged 8–9 years. Contrary to previous studies, maternal DM, pre-pregnancy overweight, and HDP were not associated with later autistic symptoms in children. However, maternal DM and pre-pregnancy overweight were found to be associated with increased leptin concentrations in cord serum and that, in turn, leptin levels were associated with autistic symptoms in total and in the DSM-5 compatible subdomains (namely, SCI and RRB). These findings suggest the importance of leptin in ASD etiology. Another important implication of the present study is that maternal metabolic conditions before or during pregnancy were not found directly associated with autistic symptoms in children, but leptin levels increased by those conditions might affect later neurodevelopment.

## Data Availability Statement

The data generated for this study is subject to the following licenses/restrictions: Privacy and confidentiality of participants. Requests to access these datasets should be directed to Kenji J. Tsuchiya, tsuchiya@hama-med.ac.jp.

## Ethics Statement

The studies involving human participants were reviewed and approved by the Hamamatsu University School of Medicine and the University Hospital Ethics Committee. Written informed consent to participate in this study was provided by the participants' legal guardian/next of kin.

## Author Contributions

TI and NT had full access to all the data used in the study and takes responsibility for the integrity of the data and accuracy of the data analysis. TI, NT, and KT conceptualized the study and drafted the manuscript. TI, NT, TN, and MR performed the statistical analyses. TN, TH, AO, and HM provided technical and material support. KT supervised the study. All authors conducted the data acquisition, contributed to critical revision of the manuscript and significantly to the study, and the creation of this manuscript.

## Funding

This work was supported by grants from the Ministry of Education, Culture, Sports, Science and Technology in Japan (Grant Number 19K14175 to TI; Grant Numbers 19H03582, 21K19639, and 21KK0145 to KT), AMED (Grant Number JP21gk0110039h0003 to KT), and the National Institute of Mental Health (Grant Number NIMH R01 MH102729 to YN).

## Conflict of Interest

The authors declare that the research was conducted in the absence of any commercial or financial relationships that could be construed as a potential conflict of interest.

## Publisher's Note

All claims expressed in this article are solely those of the authors and do not necessarily represent those of their affiliated organizations, or those of the publisher, the editors and the reviewers. Any product that may be evaluated in this article, or claim that may be made by its manufacturer, is not guaranteed or endorsed by the publisher.
