# Supplementary material for: Associations Among Maternal Metabolic Conditions, Cord Serum Leptin Levels, and Autistic Symptoms in Children
Source: Front Psychiatry. 2022 Feb 3;12:816196. doi: 10.3389/fpsyt.2021.816196 (PMC8851349; doi:10.3389/fpsyt.2021.816196)
Supplement: Supplementary file 1 [file Table_1.DOCX]

**Supplementary Table S1**. Associations among variables of interest (leptin levels and SRS-2 total T-score) and covariates in Model 1.

| Outcome | Covariate | Coefficient (95% CI)^a^ | *P*-value |
| --- | --- | --- | --- |
| SRS-2 total | Child’s sex | 1.017 (0.996 to 1.037) | 0.099 |
|  | Maternal age at delivery | 1.000 (0.998 to 1.002) | 0.533 |
|  | Maternal education | 0.995 (0.989 to 1.000) | 0.092 |
|  | Household income | 0.999 (0.999 to 1.000) | 0.092 |
|  | Gestational age | 0.993 (0.947 to 1.042) | 0.801 |
|  | Maternal smoking status | 1.004 (0.966 to 1.043) | 0.821 |
|  | Birth weight | 0.999 (0.999 to 1.000) | 0.596 |
|  | Mode of feeding | 1.004 (0.990 to 1.016) | 0.584 |
| Leptin level | Child’s sex | **0.549 (0.493 to 1.004)** | **<0.001** |
|  | Maternal age at delivery | **0.983 (0.973 to 0.995)** | **0.005** |
|  | Maternal education | 1.007 (0.975 to 1.039) | 0.665 |
|  | Household income | 0.999 (0.999 to 1.000) | 0.376 |
|  | Gestational age | **0.699 (0.538 to 0.909)** | **0.008** |
|  | Maternal smoking status | 1.100 (0.894 to 1.354) | 0.364 |
|  | Birth weight | **1.001 (1.000 to 1.001)** | **< 0.001** |
|  | Mode of feeding | 0.998 (0.929 to 1.072) | 0.961 |

^a^ Coefficients are exponentiated. Abbreviations: SRS-2, the Social Responsiveness Scale, Second Edition.
